# Frizzled-9 impairs acetylcholine receptor clustering in skeletal muscle cells

**DOI:** 10.3389/fncel.2014.00110

**Published:** 2014-04-17

**Authors:** Evelyn C. Avilés, Cristina Pinto, Patricia Hanna, Jorge Ojeda, Viviana Pérez, Giancarlo V. De Ferrari, Pedro Zamorano, Miguel Albistur, Daniel Sandoval, Juan P. Henríquez

**Affiliations:** ^1^Laboratory of Developmental Neurobiology, Department of Cell Biology, Faculty of Biological Sciences, Millennium Nucleus of Regenerative Biology, Center for Advanced Microscopy, Universidad de ConcepciónConcepción, Chile; ^2^Faculty of Biological Sciences, Center for Biomedical Research and FONDAP Center for Genome Regulation, Universidad Andres BelloSantiago, Chile; ^3^Department of Biomedicine, Universidad de AntofagastaAntofagasta, Chile

**Keywords:** Frizzled receptors, Wnt proteins, neuromuscular junction, acetylcholine receptor, postsynaptic, skeletal muscle

## Abstract

Cumulative evidence indicates that Wnt pathways play crucial and diverse roles to assemble the neuromuscular junction (NMJ), a peripheral synapse characterized by the clustering of acetylcholine receptors (AChR) on postsynaptic densities. The molecular determinants of Wnt effects at the NMJ are still to be fully elucidated. We report here that the Wnt receptor Frizzled-9 (Fzd9) is expressed in developing skeletal muscles during NMJ synaptogenesis. In cultured myotubes, gain- and loss-of-function experiments revealed that Fzd9-mediated signaling impairs the AChR-clustering activity of agrin, an organizer of postsynaptic differentiation. Overexpression of Fzd9 induced the cytosolic accumulation of β-catenin, a key regulator of Wnt signaling. Consistently, Fzd9 and β-catenin localize in the postsynaptic domain of embryonic NMJs *in vivo*. Our findings represent the first evidence pointing to a crucial role of a Fzd-mediated, β-catenin-dependent signaling on the assembly of the vertebrate NMJ.

## Introduction

At the vertebrate NMJ, extracellular matrix and signaling proteins, secreted either by the nerve or the muscle, play stimulatory, and inhibitory roles to orchestrate the assembly of functional synapses (Sanes and Lichtman, 2001; Wu et al., 2010). An early hallmark of postsynaptic differentiation at the NMJ is the aggregation of several postsynaptic proteins, including the acetylcholine receptors (AChRs), in discrete domains of the sarcolemma (Sanes and Lichtman, 2001). Before innervation, a pre-pattern of AChR clusters forms on the muscle surface to guide the subsequent positioning of motor axons for NMJ assembly (Lin et al., 2001; Vock et al., 2008; Jing et al., 2009). Upon nerve-muscle contact, most pre-patterned AChR clusters are disassembled by the inhibitory effect of acetylcholine (ACh) (Lin et al., 2005; Misgeld et al., 2005; An et al., 2010), except the ones located in close apposition with the motor axon which are stabilized by the motor neuron-derived proteoglycan agrin (Lin et al., 2005; Misgeld et al., 2005). Agrin signals through the muscle-specific tyrosine kinase receptor MuSK (Valenzuela et al., 1995; Dechiara et al., 1996; Glass et al., 1996), by forming a membrane complex with the low density lipoprotein receptor-related protein 4 (Lrp4) (Weatherbee et al., 2006; Kim et al., 2008; Zhang et al., 2008). Agrin induces AChR clustering in cultured myotubes, but its *in vivo* role is likely to prevent the inhibitory role of the neurotransmitter, thus maintaining postsynaptic specializations (Misgeld et al., 2005). Therefore, the embryonic assembly of the NMJ relies on signals that play positive and inhibitory effects on AChR clustering at the postsynaptic membrane.

In the last years, strong evidence has proven a crucial role for Wnt signaling on different features related to the connectivity and function of the nervous system. Wnt ligands -of which 19 family members are found in vertebrates- signal through their cognate seven-pass transmembrane G-protein coupled Frizzled (Fzd) receptors (10 family members found in vertebrates) (Gordon and Nusse, 2006) to activate at least three different signaling pathways. In the “Wnt canonical” pathway, the glycogen synthase kinase-3β (GSK-3β) is inhibited resulting in the intracellular accumulation of the key effector β-catenin, which then translocates to the nucleus where, along with Tcf/Lef1 transcription factors, activates the expression of specific Wnt target genes (Gordon and Nusse, 2006; Kim et al., 2009). Importantly, β-catenin also plays crucial roles in cell membrane protein complexes for cell-cell adhesion (Brembeck et al., 2006). Wnts also trigger two “non-canonical” pathways: a “Wnt calcium” pathway that regulates cell fate and cell movement by increasing intracellular Ca^2+^ levels (Kuhl et al., 2000), and the “planar cell polarity” pathway which acts locally to modify the cytoskeleton through the small Rac and Rho GTPases (McEwen and Peifer, 2000). Due to the high heterogeneity of Wnt ligands, receptors and pathways, specific responses to activation of different Wnt cascades depend on specific effectors for each cell type and biological context.

Wnt pathways play pro- and anti-synaptogenic effects to regulate the formation or distribution of the vertebrate and invertebrate NMJ (Korkut and Budnik, 2009; Wu et al., 2010; Henriquez et al., 2011; Henriquez and Salinas, 2012). Early on in the formation of the vertebrate NMJ, Wnt ligands secreted by muscle cells or the surrounding tissues have been shown to interact with MuSK to induce the aneural pre-patterning of AChR clusters on the muscle surface (Jing et al., 2009; Gordon et al., 2012). Upon NMJ synaptogenesis, Wnt ligands can play pro and inhibitory roles on postsynaptic differentiation. On the one hand, the Wnt3 ligand, which is expressed by motor neurons at the time of NMJ formation (Krylova et al., 2002), collaborates with agrin to induce AChRs clustering via a small GTPase-dependent, non-canonical Wnt signaling (Henriquez et al., 2008). On the other hand, the highly identical muscle-derived Wnt3a ligand impairs agrin-induced AChR clustering and disassemble pre-formed aggregates via a β-catenin-dependent, but TCF-independent, pathway (Wang and Luo, 2008; Wang et al., 2008). Thus, we have hypothesized that activation of different Wnt pathways could control opposite, but complementary roles on postsynaptic differentiation at nascent NMJs (Henriquez and Salinas, 2012).

In order to gain insights into the possible mechanisms employed by muscle cells to convert Wnt signals into positive or negative inputs, here we evaluated whether Fzd receptors could mediate these differential Wnt effects. Based on previous findings showing that Fzd9 expression is modulated by innervation in skeletal muscle (Magnusson et al., 2005) and due to its role in the formation of neuronal connectivity (Zhao and Pleasure, 2004; Zhao et al., 2005), we focused on the expression pattern and possible function of this Wnt receptor on postsynaptic differentiation at the NMJ.

## Experimental procedures

### Animals

Swiss Webster mice were obtained from Animal Facilities at the Faculty of Biological Sciences, Universidad de Concepción. Experiments were conducted following the guidelines outlined in the Biosafety and Bioethics Manual of the National Commission of Scientific and Technological Research (CONICYT, Chilean Government). The Bioethics Committee of Universidad de Concepción (Concepción, Chile) approved all experimental procedures carried out during this study.

### Reverse transcription-PCR

Total RNA was extracted from hemidiaphragms of E14.5, E19.5, and 6-week old mice, as well as from differentiated myotubes from the C2C12 cell line, using Trizol reagent (Invitrogen, Carlsbad, CA, USA), following the indications of the manufacturer. For RT-PCR, 1 μg of RNA was pre-treated with DNase I (Fermentas, Ontario, Canada) and further incubated in a buffer containing 5 μM oligo dT primer, reverse transcription buffer (0.5 M Tris-HCl, pH 8.3, 0.75 M KCl, 0.03 M MgCl_2_), 20U RNase inhibitor (NEB, Ipswich, MA, USA) and 0.5 mM dNTPs at 37°C for 5 min. A volume to reach 160 U Stratascript reverse transcriptase (Stratagene, La Jolla, CA, USA) was added, and the mix was further incubated at 42°C for 1 h. Parallel reactions were performed in the absence of reverse transcriptase to control for the presence of genomic DNA. For amplification, a RT aliquot in 75 mM Tris buffer, pH 8.8, 20 mM (NH_4_) SO_4_, 0.01% Tween 20, 1.5 mM MgCl_2_, 0.2 mM dNTPs, 0.2 mM primers, and 0.35 U Taq polymerase (Fermentas) was subjected to 30 cycles of PCR. The annealing temperatures were set as indicated in Table 1. Primers were designed to amplify specific, non-conserved regions of Fzd receptors (Table 1). A 233 bp fragment of mouse GAPDH mRNA was also amplified as housekeeping control.

**Table 1:** **List of primers used to detect the mRNA expression by RT-PCR of Frizzled 1–10 and the housekeeping control GAPDH**.

**Gene**		**Sequence**	**Tm (°C)**	**Ta (°C)**	**Product size (bp)**
Fz1	S	CTCTTCCGCATCCGCACC	59	55	336
	AS	GCCGGACCAGATCCGGAA	60		
Fz2	S	CCCGGGCGGCCCTGGCGG	74	45	271
	AS	GTCCACTAAATAGGTGGT	48		
Fz3	S	CCACTCAAGGGACATCCA	54	50	407
	AS	AGCCAGCCATGCGAAGGC	62		
Fz4	S	TGGCTTIGTGGIGGCTCC	58	54	474
	AS	AGGAACGAGGAAGCCGGC	60		
Fz5	S	GCGCACCGGCCAAGTGCC	66	58	270
	AS	CGGCTGCAAGCGACGCTG	63		
Fz6	S	GGCAATCGCTGACCATGA	56	51	308
	AS	TGGCGGCCTGTGAAGTGC	62		
Fz7	S	CGGTGCCGGCCACCATCG	65	56	403
	AS	GTGGAGGGGGCAGGTAGC	61		
Fz8	S	CCGTGCTCTACACGGTGC	59	58	276
	AS	CTGCAGCGCCCTTGCTGG	63		
Fz9	5	GCTGGAGAAGCTGATGGT	55	50	437
	AS	CCAGAGAGGGGTCTGTCT	56		
Fz10	S	TGTGCCGGCCACCTGTGTGATTGC	66	61	246
	AS	ACGTOTTOCCAGGACTGCAGGGTC	66		
GAPDH	S	GGAGCCAAACGGGTCATCATCTC	60	55	233
	AS	GAGGGGCCATCCACAGTCTTCT	61		

The melting (Tm) and annealing (Ta) temperatures are indicated as well as the sequences and the size expected for the PCR products.

### Plasmids

The DNA sequence coding for mouse Fzd9 was amplified from a previously cloned vector (a gentle gift of Dr. Robert Winn, University of Colorado, CO, USA). The HA tag was fused to the C-terminal end of Fzd9 by overlapping PCR, and the resulting sequence (Fzd9HA) was subsequently cloned between the XhoI and XbaI sites of the pCS2+ expression vector. A similar strategy was followed to clone mouse Wnt2, in which the full-length coding sequence was amplified from total RNA obtained from adult heart. To inhibit Fzd9 expression, we identified a potential RNA interfering sequence for mouse Fzd9 through the Dharmacon algorithm (www.dharmacon.com). A short-hairpin DNA sequence specific for Fzd9 (shFzd9) was subcloned between the BsrGI and NheI sites of the FUXH1Off-EGFP plasmid, which drives the transcription of short sequences under the H1 promoter along with expressing EGFP under the CMV promoter.

### Western blot

Mixtures of skeletal muscles from mouse hind limbs at different developmental stages (E14.5 to P0) were disrupted using a vibra cell sonicator (Sonics, Newtown, CT, USA) with five pulses at 70% power in a solution containing 0.3 M sucrose and a protease inhibitor cocktail (80 μM aprotinin, 1.5 mM pepstatin A, 2 mM leupeptin, 104 mM AEBSF, 4 mM Bestatin, 1.4 mM E-64) (Sigma, St. Louis, MO, USA). Supernatants containing the total protein extracts were obtained after centrifuging at 8000xg for 10 min at 4°C. In the case of transfected C2C12 myotubes and HEK293 cells, the samples were rinsed with PBS, harvested and homogenized in the above mentioned solution, and centrifuged for 10 min at 13,000xg and 4°C. The supernatant corresponds to the pool of cytosolic soluble proteins. The resulting pellet was resuspended in a detergent-containing buffer (0.05 M Tris-HCl, pH 7.4, 0.5% Triton X-100, 0.15 M NaCl) and centrifuged under the same conditions to obtain a membrane-enriched protein fraction. For immunoblotting, 20 μg of proteins were loaded in each lane and fractionated by PAGE-SDS, transferred to nitrocellulose membranes, and incubated with a highly specific goat anti mouse Fzd9 antibody, which does not cross-react with other related Fzd receptors (R and D Systems, Minneapolis, MN, USA). We also used goat anti human β-actin, mouse anti human β-catenin (Santa Cruz Biotechnology, Santa Cruz, CA, USA) and mouse anti chicken α-tubulin (Sigma) antibodies. Primary antibodies were incubated in 5% non-fat milk blocking buffer for 12–18 h at 4°C, whereas peroxidase-conjugated secondary antibodies (Jackson Immuno Research, West Grove, PA, USA) were incubated for 2 h at room temperature. The signal was developed by enhanced chemiluminiscence using the ECL Western blotting analysis system (Perkin Elmer, Waltham, MA, USA). The exposure time was adjusted to reveal the less abundant band (i.e., E19.5) in order to detect possible differences with the other samples in the gel. The intensities of the resulting bands were calculated with Image J software and correspond to the average ± s.e.m. of three independent experiments.

### Immunochemistry

The diaphragm muscle of E17.5 and P0 mice was removed and fixed with 4% paraformaldehyde for 1 h at 4°C and subsequently permeabilized with 0.5% Triton X-100, incubated with 0.15 M glycine, pH 7.4, for 15 min and then with 10 mg/ml NaBH_4_ for 5 min. Muscles were blocked in 0.5% Triton X-100, 0.025% BSA, 0.2% horse serum for 12–16 h at 4°C. Primary antibodies were incubated in blocking solution for 16 h at 4°C and were then washed in PBS containing 0.1% Triton X-100. Transfected myotubes were fixed with 4% paraformaldehyde for 20 min at room temperature, permeabilized with 0.1% Triton X-100 for 10 min and subsequently incubated with primary antibodies diluted in blocking solution (1% BSA in Tris phosphate buffer) for 12–15 h at 4°C. Antibodies used were goat anti mouse Fzd9 (R and D Systems) as well as mouse or rabbit anti human β-catenin (Santa Cruz Biotechnology). Corresponding Alexa488 and Alexa546-conjugated secondary IgGs (Invitrogen) were incubated for 2 h at room temperature, along with 2 ng/μL Alexa546- or Alexa647-conjugated αBTX (Invitrogen) to reveal AChR clusters. Nuclei were counterstained with ToPro-3 or DAPI (Invitrogen). Images were acquired with a laser confocal Nikon D-Eclipse C1 or a Zeiss LSM700 microscope at the CMA Bio-Bio facility at the Universidad de Concepción. The average fluorescence intensity of β-catenin in transfected myotubes was measured using Metamorph software. Data are expressed as the average of the relative value ± s.e.m. of three independent experiments.

### C2C12 cells culture and transfection

C2C12 cells were grown on glass coverslips in DMEM medium containing 10% fetal bovine serum, 2 mM L-glutamine and penicillin/streptomycin. After 24 h, myoblasts were transfected with a lipofectamine-PLUS mixture (Invitrogen) according to the instructions of the manufacturer. The DNA/lipofectamine/PLUS ratio in the mixture was 0.5 μg/1.5 μl/1 μL with 0.4 μg plasmid DNA per well. Cells were incubated with the DNA mixture and lipofectamine-PLUS in serum-free medium for 6 h before being switched to the fusion medium (DMEM containing 2% horse serum). After 5 days, fused myotubes were treated with 200 pM neural agrin (R and D Systems) for 12–18 h at 37°C. Cells were subsequently washed and fixed in 4% paraformaldehyde for 20 min at room temperature. Fzd9-transfected cells were stained with a goat anti-Fzd9 (Invitrogen) or a rat anti-HA antibody (Roche Applied Science, Mannheim, Germany). AChR clusters were stained with 2 ng/μL Alexa546-conjugated αBTX (Invitrogen), along with an Alexa488-conjugated secondary antibody (Invitrogen) for 2 h at room temperature.

### Image analyses

Fluorescent images were acquired with a laser confocal Nikon D-Eclipse C1 microscope in *z* series of 2 μm intervals to cover the entire depth of myotubes. Stack and 2D deconvoluted images were obtained using Metamorph software. The surface of transfected myotubes (seen in the green channel) was manually traced and their area was measured using Metamorph. The area of each AChR cluster larger than 4.5 μm^2^ present on transfected myotubes was automatically determined using the same software. Data are expressed as total area of AChR clusters (μm^2^) per mm^2^ of transfected myotube area, total average of AChR clusters (μm^2^/mm^2^ of myotube) and average area of AChR clusters (μm^2^).

### Luciferase assays

For luciferase assays, the amounts of plasmid DNAs used for trasfections of HEK293 cells were: 20 ng of the Wnt reporter Top Flash, 2 ng of the control Renilla reporter, 150 ng Wnt2, 75 ng Fzd9, and 150 ng shFzd9 per well of a 24-well plate. When required, empty pCS2+ or FUXH1Off-EGFP plasmids were added in a sufficient amount to reach 0.4 μg of total plasmid DNA per well. After 48 h, cells were lysed and the activity of the Topflash and Renilla reporter genes was measured using the Dual Luciferase Report Assay System (Promega, Madison, WI, USA) according to the indications of the manufacturer. Data are expressed as the Topflash: Renilla ratio, relative to the basal background activity obtained from cells transfected with control plasmids.

### Statistical analyses

Plots correspond to the average ± s.e.m. of three independent experiments performed by triplicate, except when indicated. Data were statistically analyzed using non-parametric *t*-test or ANOVA, as indicated in the figure legends.

## Results

**Figure 1:**
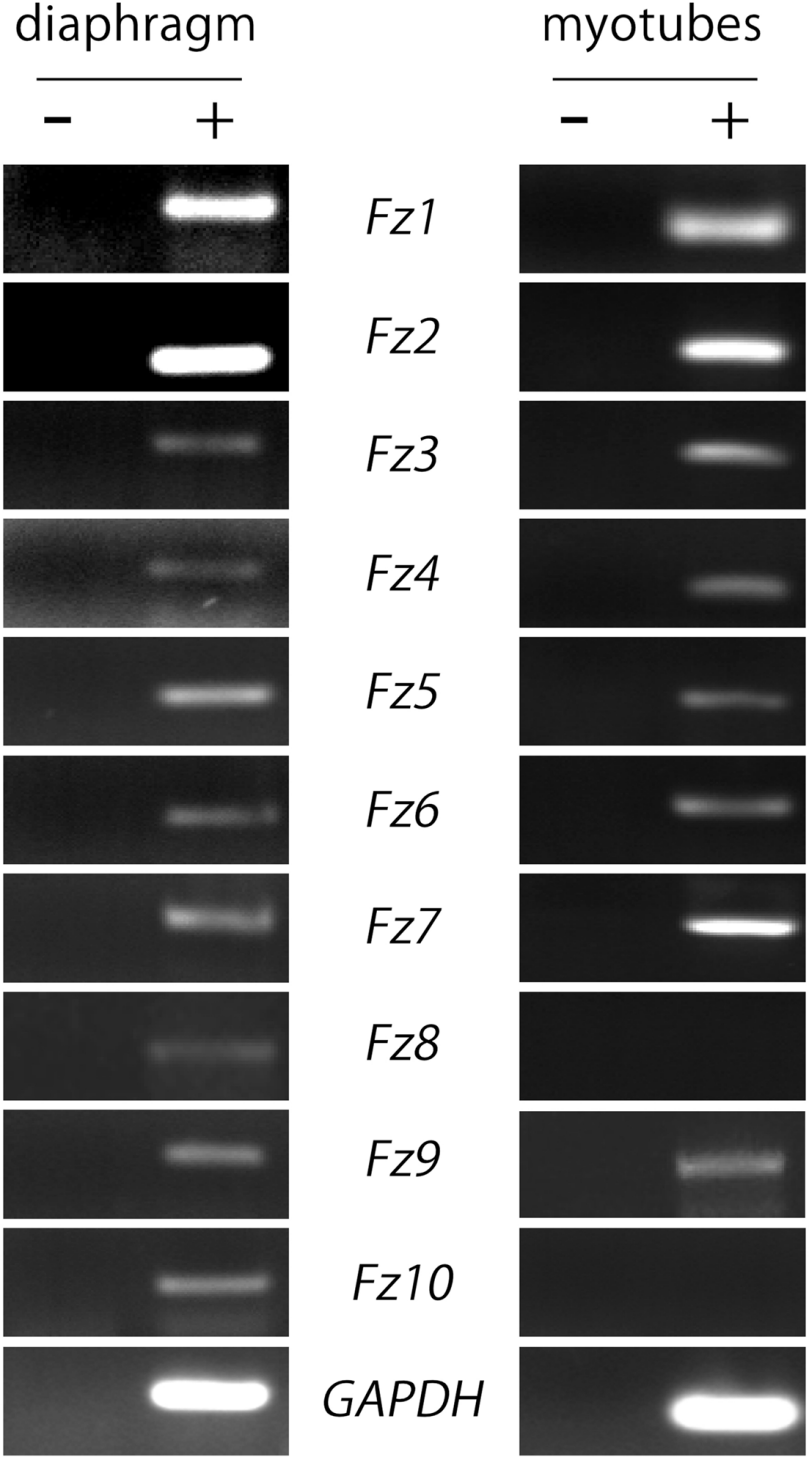
**Most *Fzd* receptors are expressed in skeletal muscle cells**. Total RNAs were extracted from 6-week old mouse diaphragm and cultured myotubes and further subjected to RT-PCR to detect the mRNA expression of all ten mouse *Fzd* receptors (+). As a negative control, samples were processed in the absence of reverse transcriptase (−). *Fzd* receptors 1 to 10 are expressed in the mouse diaphragm (*left panel*). In cultured C2C12 myotubes, almost all Fzd receptors are expressed at the mRNA level, except for Fzd8 and Fzd10, which were not amplified by RT-PCR (*right panel*). GAPDH expression was used as a loading control gene. Gels are representative of at least three experiments performed by triplicate.

As a first hint to examine the potential contribution of Fzd-mediated signaling on postsynaptic development at the NMJ, we analyzed the expression pattern of the different Fzd receptors in muscle cells. We obtained total RNA extracts from 6-week old mice hemidiaphragms and from cultured myotubes of the C2C12 muscle cell line to perform RT-PCR analyses. We used specific primers (Table 1) to amplify internal fragments of the ten mice Fzd receptors. As shown in Figure 1, fragments of the expected molecular sizes were amplified from mice muscles for the ten Fzd mRNAs. A similar result was obtained from C2C12 myotubes, except for Fzd8 and Fzd10 transcripts, which were not detected. A 233 bp fragment of mouse GAPDH mRNA was amplified as housekeeping control. Negative control experiments performed in the absence of reverse transcriptase were not amplified.

**Figure 2:**
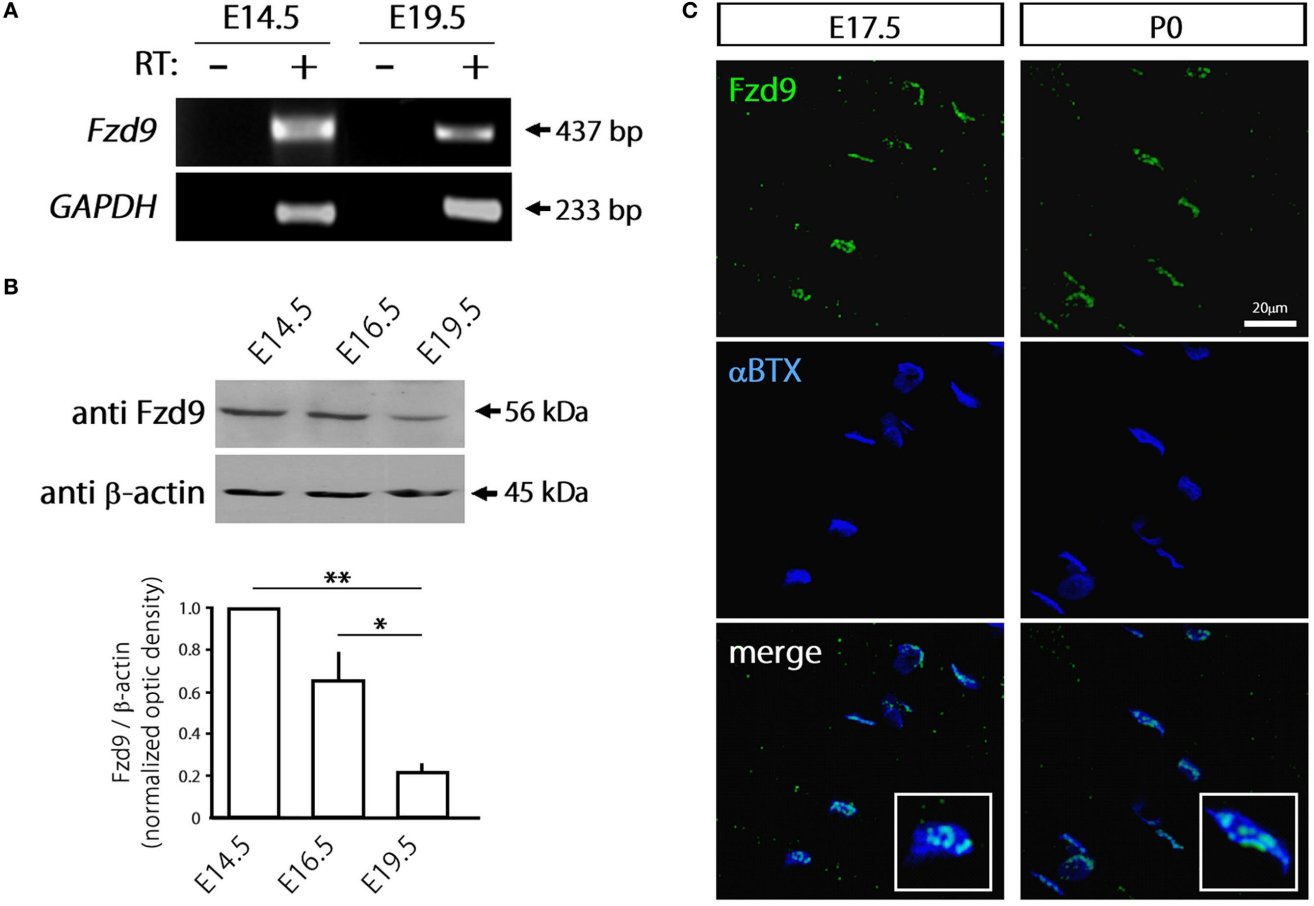
**Fzd9 is dynamically expressed in skeletal muscle tissue. (A)** Total RNAs were extracted from E14.5 and E19.5 mouse hind limb muscles and further subjected to RT-PCR to detect the mRNA expression of *Fzd9* (+). As a negative control, samples were processed in the absence of reverse transcriptase (−). GAPDH expression was used as a loading control gene. The gel is representative of two experiments performed by triplicate. **(B)** Proteins from mouse hind limb skeletal muscles obtained at different developmental stages (E14.5, E16.5, and E19.5) were fractionated by SDS-PAGE and immunoblotted for Fzd9 and β-actin (*upper panel*). Fzd9 is expressed at the protein level in all the analyzed developmental stages since the expected 56kDa band is detected. A representative gel shows that Fzd9 band intensity displays a progressive decrease from E14.5 to E19.5. Data represent the mean ± *SD* of Fzd9/β-actin ratio from three experiments, normalized to E14.5 (*lower panel*) (^*^p < 0.05, ^**^p < 0.01, ANOVA using Bonferroni's *post-hoc* analysis). **(C)** Whole-mounted diaphragms from E17.5 and P0 were stained with anti Fzd9 antibody (green) together with αBTX to reveal the postsynaptic densities (blue). Fzd9 is abundant in the synaptic domain of embryonic NMJs, where it displays a punctate expression pattern. The insets (*lower panels*) show that some αBTX-positive regions were not labeled with anti Fzd9 antibodies. Pictures are representative of at least three experiments performed by triplicate.

Considering that most Fzd receptors are likely expressed by skeletal muscle cells, we focused on Fzd9 based on previous findings suggesting a relevant role for this Wnt receptor on nerve-muscle communication in 6-week old mice (Magnusson et al., 2005), as well as on the formation and function of central synapses (Zhao and Pleasure, 2004; Zhao et al., 2005). In order to assess a potential role for Fzd9 on the formation of the NMJ, we first analyzed the expression pattern of Fzd9 in embryonic skeletal muscle tissue (Figure 2). RT-PCR analyses show that the fragment amplified with specific primers for Fzd9 was diminished in E19.5 as compared to E14.5 (Figure 2A). Consistently, immunoblots of developing mouse skeletal muscles showed relatively high expression of Fzd9 at early stages of NMJ assembly (E14.5) and gradually decreases as development proceeds, being more than ~4-fold less expressed in E19.5 (Figure 2B). Thus, the temporal expression of Fzd9 in muscle supports a potential role for this Wnt receptor on neuromuscular synaptogenesis. To analyze the distribution of Fzd9 at the developing NMJ, we performed immunohistochemical studies in whole-mounted hemidiaphragms obtained from E17.5 and P0 mice. Our findings show that Fzd9 staining was intense in discrete domains of the sarcolemma at both developmental stages (Figure 2C). To analyze whether the Fzd9-enriched regions corresponded to synaptic domains, we double stained with fluorescently-conjugated α-bungarotoxin (αBTX), which binds with high specificity to AChR aggregates (Sanes and Lichtman, 2001). Figure 2C shows that postsynaptic muscle domains contain high Fzd9 labeling, which distribute in a punctate pattern. Indeed, magnified pictures (insets in Figure 2C) show that this co-distribution pattern is rather incomplete, as αBTX-positive regions were not labeled with anti Fzd9 antibodies. Overall, the temporal expression and distribution of Fzd9 support a possible role for this Wnt receptor during NMJ assembly.

**Figure 3:**
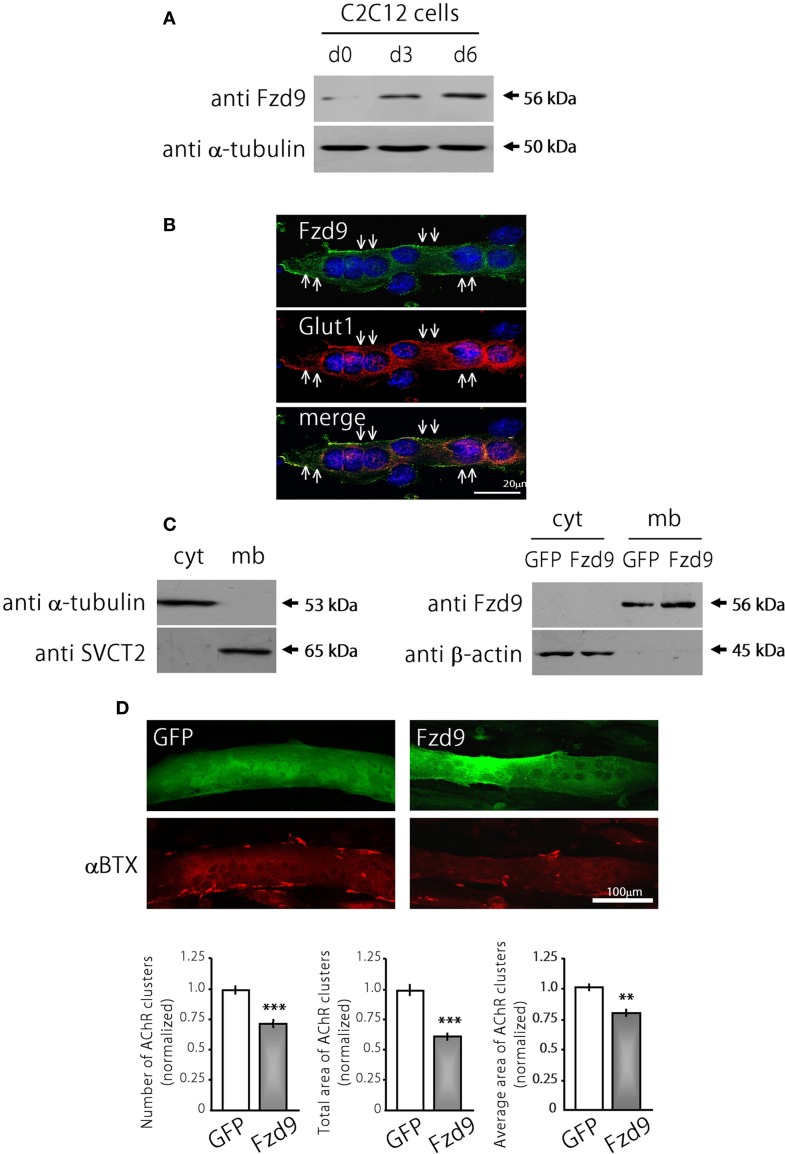
**Fzd9 impairs agrin-dependent AChR clustering in myotubes. (A)** Fzd9 is expressed in the muscle cell line C2C12 throughout differentiation. C2C12 cells were cultured *in vitro* and differentiated for 0, 3, or 6 days (d0-d6). Total proteins were subjected to Western blot analyses. An expected 56 kDa band is gradually increased during C2C12 cells differentiation. α-tubulin expression was used as a loading control. **(B)** C2C12 myotubes differentiated for 6 days were analyzed by immunocytochemistry to detect Fzd9. Fzd9 is localized to the plasma membrane of the myotubes (green, *upper panel*), similar to Glut1, which was used as a marker of plasma membrane (red, *middle panel*). The merge image (*lower panel*) reveals the co-localization of Fzd9 and Glut1. **(C)** Differentiated C2C12 myotubes were subjected to a sequential fractionation procedure to isolate samples enriched in cytoplasm (cyt) or plasma membrane (mb) proteins. Western blot analyzes showed that α-tubulin is specifically detected in cytoplasmic fractions, whereas the vitamin C transporter SVCT2 was only present in membrane-enriched protein fractions (*left panel*). C2C12 myoblasts were transfected either with GFP or Fzd9 and differentiated. Sequential protein lysates were analyzed by Western blot. Both endogenous Fzd9 (GFP-transfected cells) or overexpressed Fzd9 (Fzd9-transfected cells) were found predominantly in the plasma membrane and were absent in the cytoplasm. As a loading control, β-actin is found only in the cytoplasm-enriched fraction (*right panel*). **(D)** Myoblasts transfected with plasmids coding for GFP (control) or Fzd9 were differentiated into myotubes and subsequently incubated with 200 pM neural agrin. αBTX staining allows the visualization of the AChRs (red). Automatized quantification of aggregates shows that Fzd9 overexpression induces a decrease in the number of AChR clusters per myotube, as well as a reduction in the total area and average size of AChR clusters, compared to controls. Data represent the mean ± s.e.m. (*n* = 3 performed by triplicate; normalized to GFP-transfected myotubes). (^**^p < 0.01, ^***^p < 0.001 compared to GFP controls, *t*-test).

**Figure 4:**
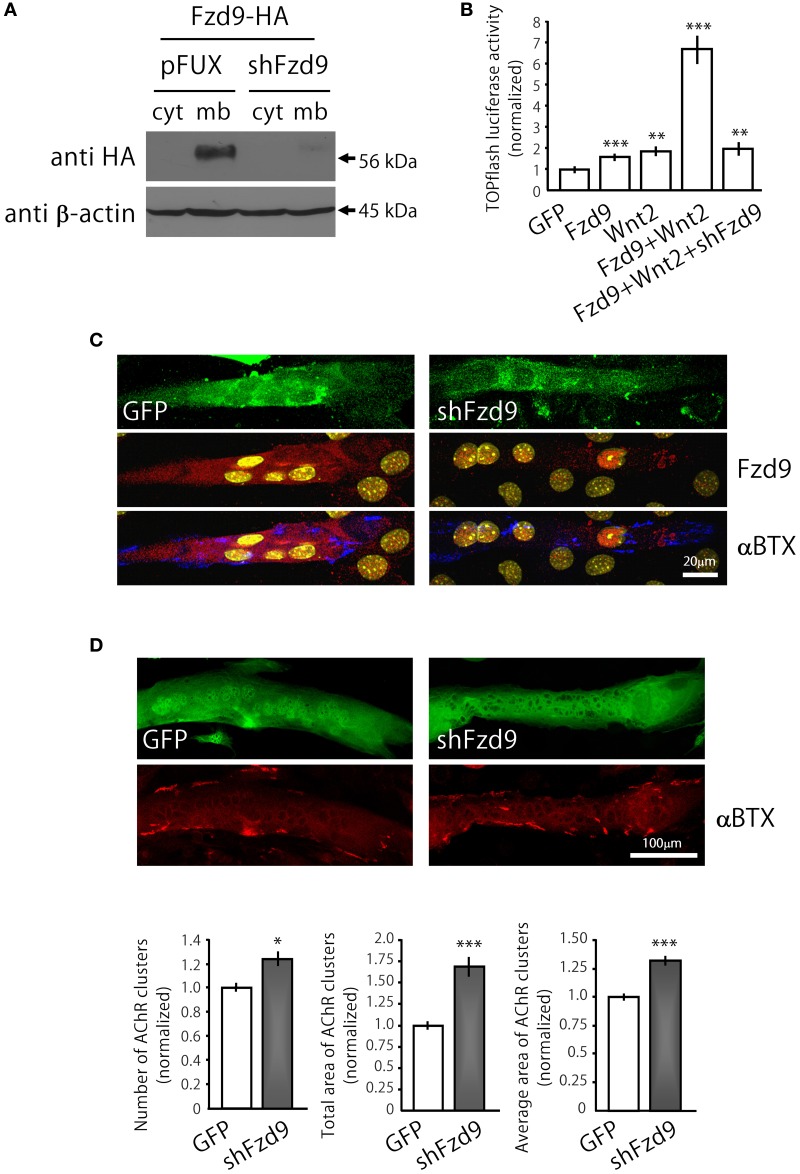
**Down-regulation of Fzd9 increases agrin-dependent AChR clustering in myotubes. (A,B)** The efficiency of shFzd9 was tested by its ability to impair overexpression/function of the Fzd9 construct. **(B)** HEK293 cells were transfected with Fzd9HA together with a control shRNA (pFUX) or shFzd9, followed by protein homogenization and Western blot. Whereas a 56 kDa band corresponding to Fzd9 is detected in the membrane-enriched fraction of the control condition, Fzd9 expression is drastically silenced in cells transfected with shFzd9. **(B)** The efficiency of the shFzd9 to affect the functionality of Fzd9HA was assessed by co-transfecting Fzd9HA and Wnt2 in the presence or absence of the shFzd9 plasmid in HEK293 cells. Activation of the TOPflash luciferase reporter gene was used as a readout of activation of the canonical Wnt pathway. These experiments were performed at least three times by triplicate (^**^p < 0.01, ^***^p < 0.001, *t*-test). **(C)** Myoblasts were transfected either with GFP or shFzd9 and differentiated for 5 days. Myotubes were treated with neural agrin and further stained with an anti-Fzd9 antibody (red), along with αBTX (blue) to detect AChR clusters and DAPI (yellow) to stain nuclei. shFz9-transfected myotubes display silenced Fzd9 expression and an apparent increase in the number of AChR clusters compared to GFP-expressing myotubes. **(D)** Myoblasts transfected either with GFP or shFzd9 and grown for 5 days were treated with neural agrin and further stained with αBTX to detect AChR clusters (red). Myotubes expressing the GFP protein present in the shFzd9 plasmid show a significant increase in the number of AChR clusters, an increase in the total area of AChR clusters, as well as on the average size of AChR clusters, when compared to control myotubes that only express GFP. Data represent the mean ± s.e.m. (*n* = 3 performed by triplicate; normalized to GFP-transfected myotubes). (^*^p < 0.05, ^***^p < 0.001 compared to GFP controls, *t*-test).

The possible function of Fzd9 on postsynaptic assembly at the NMJ was examined in cultures of the C2C12 muscle cell line. The expression of Fzd9 in myoblasts (day 0 of differentiation) is very low and becomes much higher as differentiation proceeds at day 3 and day 6 (Figure 3A). The endogenous Fzd9 protein is localized to the plasma membrane of myotubes (arrows in Figure 3B), where it exhibits a similar pattern as the glucose transporter Glut1. In order to perform gain-of-function experiments, we cloned the coding region of mouse Fzd9, fused to the HA tag, into the pCS2+ expression vector. In order to validate the resulting Fzd9HA plasmid, we performed control experiments. First, C2C12 myoblasts were transfected with Fzd9HA, or with the control empty plasmid, and differentiated for 5 days. We then performed a sequential subcellular fractionation to separate cytosolic- and membrane-enriched fractions. Control Western blot experiments show that α-tubulin is present only in the cytosolic fraction, whereas the sodium/vitamin C co-transporter SVCT2 (Sandoval et al., 2013) was specifically detected in the membrane fraction, thus demonstrating the efficiency of the fractionation protocol (Figure 3C, *left panel*). Following this approach, we observed that most Fzd9 is extracted in a membrane-enriched protein fraction after differential separation of myotube proteins, while β-actin is concentrated in the cytosolic fraction (Figure 3C, *right panel*). Then, as we obtained relatively low transfection efficiency in mouse muscle-derived C2C12 cells (~10–15%), the ability of Fzd9 to transduce Wnt signaling was assayed in the human kidney-derived HEK293 cells (Figure 4B). In these highly transfectable (>90%) cells, Fzd9HA was able to mediate the Wnt2-dependent activation of a Wnt/β-catenin reporter gene, as it had been previously demonstrated for Fzd9 (Karasawa et al., 2002). Together, these data demonstrate the efficiency of the Fzd9HA tool to induce Fzd9 expression and function.

We then analyzed transfected C2C12 myotubes to determine if Fzd9 could modulate AChR clustering, an early hallmark of postsynaptic differentiation at the NMJ (Sanes and Lichtman, 2001). With this aim, control GFP and Fzd9HA-transfected myotubes were treated with neural agrin to induce AChR clusters, which were subsequently stained with αBTX, and quantified (Henriquez et al., 2008). Myotubes expressing Fzd9HA displayed a marked decrease of agrin-induced AChR aggregation, expressed here as total area of AChR clusters (25.36 × 10^3^ ± 1.3 × 10^3^ μm^2^ per mm^2^ in control GFP-transfected cells vs. 15.00 × 10^3^ ± 0.9 × 10^3^ μm^2^ per mm^2^ in Fzd9-transfected myotubes, *p* < 0.001), and a significant reduction in cluster average size (16.6 ± 0.6 μm^2^ in control GFP-transfected cells vs. 14.3 ± 0.5 μm^2^ in Fzd9-expressing myotubes) (Figure 3D). These data reveal that the overexpression of Fzd9 impairs the ability of muscle cells to aggregate AChRs.

In order to analyze the contribution of endogenous Fzd9 to agrin-mediated AChR clustering, we generated the shFzd9 plasmid, which drives the transcription of a Fzd9 RNA interference sequence under the control of the H1 promoter, plus EGFP under the control of the CMV promoter to check for transfection efficiency. Control experiments in co-transfected HEK293 cells showed that shFzd9 transfection was able to abolish the expression of Fzd9HA (Figure 4A), and inhibited the ability of Fzd9HA to mediate the Wnt2-dependent activation of Wnt/β-catenin signaling (Figure 4B). In C2C12 myotubes, qualitative analyses showed that myotubes transfected with shFzd9 display reduced Fzd9 staining and increased number of AChR clusters as compared to control GFP-expressing myotubes (Figure 4C), suggesting that postsynaptic differentiation is up-regulated upon Fzd9 silencing. In order to quantify these results, AChR clusters were analyzed in myotubes bearing green fluorescence (Figure 4D). Quantification of the data shows that Fzd9 silencing by shFzd9 transfection resulted in an increase in the number and size of AChR clusters than those on the surface of control myotubes transfected with the GFP-expressing plasmid (Figure 4D). The total number of AChRs per myotube was increased by ~23% in myotubes with silenced expression of Fzd9, whereas the total area of AChR clusters was significantly increased by ~68%, compared to the control group. The size of AChR clusters was incremented by ~32% in myotubes transfected with shFzd9. Therefore, Fzd9 inhibition enhanced the ability of agrin to induce AChR clustering. Altogether, these *in vitro* data support a specific role for Fzd9 on postsynaptic differentiation at the vertebrate NMJ.

**Figure 5:**
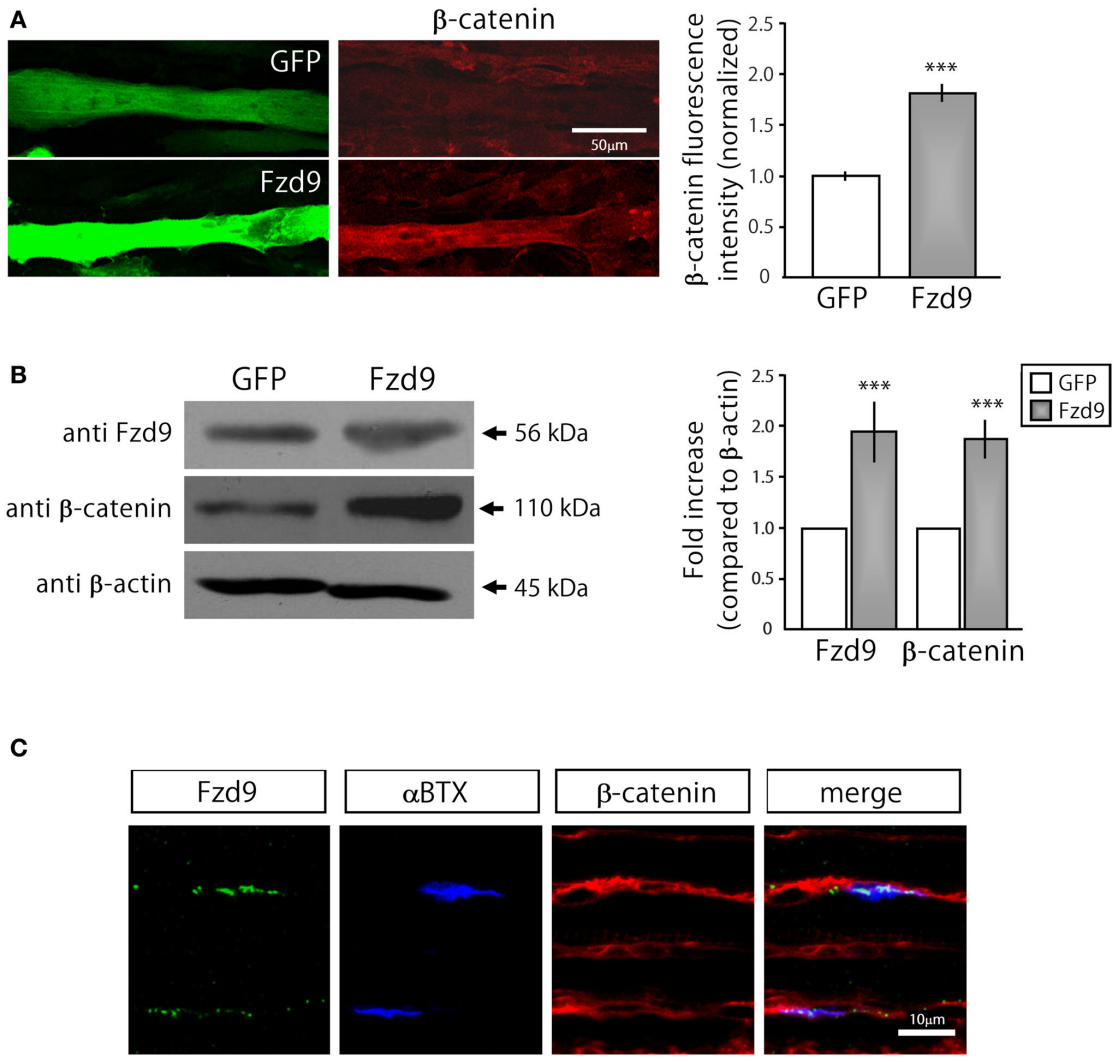
**Fzd9 enhances β-catenin accumulation in myotubes. (A)** GFP and Fzd9-transfected myotubes were immunostained with an anti β-catenin antibody (red). Fluorescence intensity of transfected myotubes was quantified using Metamorph. Quantification of the data (*right panel*) shows that the expression of Fzd9 induces a significant ~2-fold accumulation of β-catenin in the sarcoplasma, compared to control GFP-expressing myotubes. **(B)** Total protein samples from GFP- and Fzd9-transfected myotubes were separated by SDS-PAGE and immunoblotted with Fzd9 and β-catenin antibodies. Quantification of the Fzd9 or β-catenin against β-actin band intensity ratios shows that Fzd9-overexpressing myotubes display a ~2-fold increase in β-catenin cytosolic levels, which is equivalent to the ~2-fold increase observed for Fzd9 levels, compared to control myotubes (*right panel*). Data represent the mean ± s.e.m. (*n* = 3 performed by triplicate; normalized to control GFP cells; ^***^p < 0.001, *t*-test, compared to the GFP group). **(C)** Whole-mounted diaphragms of E17.5 mice were immunostained to detect Fzd9 (green) and β-catenin (red). AChR aggregates were stained with αBTX (blue). β-catenin is associated to the sarcolemma of embryonic muscle fibers, including the membrane domains where AChR clusters and Fzd9 are localized. Pictures are representative of at least three experiments performed by triplicate.

Based on previous findings indicating that AChRs dissagregate through a β-catenin-dependent pathway (Wang and Luo, 2008; Wang et al., 2008), we next analyzed whether Fzd9 overexpression in myotubes resulted in β-catenin accumulation (Figure 5). Indeed, myotubes transfected with Fzd9 showed a significant 2-fold increase in cytosolic β-catenin, evaluated by immunocytochemistry and immunoblot, when compared with control GFP-expressing myotubes (Figures 5A,B). When analyzed on E17.5 diaphragms by immunostaining, β-catenin is expressed all along the sarcolemma of muscle fibers, including the synaptic region, where AChRs are clustered and Fzd9 is localized (Figure 5C). Taken together, our present findings suggest that Fzd9 signals through a Wnt/β-catenin pathway to inhibit agrin-induced AChR clustering to shape the postsynaptic domain of the vertebrate NMJ.

## Discussion

Even though it has become clear that Wnt signaling plays pivotal roles during NMJ assembly, the identity of the receptors involved in these differential effects has remained elusive. In this regard, although Wnt ligands play pro- and anti-synaptogenic effects during NMJ assembly of invertebrate species, these opposite responses are both mediated by Fzd receptors (Mathew et al., 2005; Klassen and Shen, 2007). In turn, at the zebrafish NMJ, it is the agrin receptor MuSK, possibly via a non-canonical pathway, the one that transduces the signal of Wnt11r to induce the aneural pre-patterning of AChR clusters, an early event of postsynaptic differentiation at the vertebrate NMJ (Jing et al., 2009). Based on previous evidence showing differential roles for Wnt ligands on AChR clustering (Henriquez et al., 2008; Wang et al., 2008), in this study we analyzed the potential contribution of Fzd receptors on postsynaptic differentiation at the vertebrate NMJ. Our primary findings showed that most Fzd receptors are expressed by growing and/or differentiated muscle cells. We performed these analyses in 6-week old mice based on previous studies addressing a potential role for Wnt signaling in nerve-muscle connectivity at this time point (Magnusson et al., 2005). Interestingly, these studies revealed that Fzd9 mRNA is transcribed in adult skeletal muscles and it is significantly decreased upon muscle denervation, suggesting that the expression of muscle Fzd9 is positively regulated by its neuronal counterpart (Magnusson et al., 2005). Functionally, Fzd9 regulates the connectivity of central synapses. Indeed, Fzd9 is selectively expressed in cortical precursors and hippocampal neurons (Zhao and Pleasure, 2004; Zhao et al., 2005) and its deletion results in severe neuroanatomical defects that are functionally manifested with visuospatial learning and memory disabilities (Zhao et al., 2005). Consistent with these findings, the Fzd9 gene is deleted in the Williams syndrome, a developmental cognitive disorder in humans characterized by strong behavioral phenotypes (Wang et al., 1997, 1999). Based on this evidence, we decided to focus our subsequent analyses on the potential role that Fdz9 could play on NMJ synaptogenesis at relevant stages of embryonic development.

In our present studies, we show that the expression of Fzd9 in skeletal muscle varies as development proceeds, displaying relatively high expression at early NMJ development to subsequently decrease toward birth. Remarkably, a similar expression profile has been described for other muscle proteins that positively and negatively influence AChR clustering, such as the agrin receptor MuSK and Wnt3a, respectively (Valenzuela et al., 1995; Ip et al., 2000; Wang et al., 2008). Our findings also show that Fzd9 is specifically distributed in the postsynaptic domain of embryonic mice muscle fibers, as evidenced by its co-distribution with AChR-enriched areas. Together, the temporal expression of Fzd9 supports a potential role on embryonic NMJ assembly *in vivo*.

Our functional *in vitro* approach shows that Fzd9-mediated signaling inhibits the agrin-mediated clustering of AChR receptors. Such negative signals for AChR clustering are likely to be required not only at extrasynaptic regions of the developing embryonic NMJ, but also in specific domains of the innervated muscle region, where AChR aggregates become disassembled to shape the mature pretzel-like NMJ (Marques et al., 2000; Bolliger et al., 2010). Thus, our results on the expression profile of Fzd9 *in vivo* are consistent with its function in cultured myotubes. What is the mechanism by which Fzd9 inhibits AChR clustering? In this regard, neural- and muscle-derived molecules have been described to play such inhibitory roles at the vertebrate NMJ. One the one hand, the neurotransmitter ACh displays an AChR-disaggregating activity at the most abundant non-innervated (“extrasynaptic”) domains of the muscle membrane at nascent NMJs (Lin et al., 2005; Misgeld et al., 2005). ACh acts through the cyclin-dependent kinase 5 (Cdk5) which phosphorylates the intermediate filament protein nestin, that becomes dissociated from the cytoskeletal network and is subsequently degraded (Fu et al., 2005; Yang et al., 2011). In addition to nestin, the local dynamics of other components of the cytoskeleton also play an important role on the formation of AChR-free areas at the NMJ. For instance, punctate areas of the postsynaptic membrane, comparable to the ones we found for Fzd9, are highly enriched in polymerized F-actin by the action of ADF/cofilin. Remarkably, these areas are subsequently converted into regions devoid of AChR aggregates as development proceeds (Lee et al., 2009). Whether the punctate pattern of Fzd9 at the developing neuromuscular synapse is related to the formation of AChR-free areas for NMJ maturation is still to be elucidated.

Experiments in cultured myotubes showed that Wnt3a, which is expressed by skeletal muscles during NMJ formation, inhibits agrin-induced AChR clustering and disperses pre-formed aggregates (Wang et al., 2008). Our present findings show that agrin-induced AChR aggregation was altered by manipulation of Fzd9 expression without the exogenous addition of any Wnt ligand. Therefore, we speculate that Fzd9 could be the functional receptor of a muscle-secreted Wnt that signals to inhibit the agrin-dependent clustering of AChRs. Since Wnt3a is likely to shape the NMJ by inhibiting AChR clustering (Wang et al., 2008), it is tempting to speculate that Fzd9 could be the receptor for this Wnt. However, since aneural muscles develop more AChR clusters than controls (Lin et al., 2001), and Fzd9 expression is regulated by innervation (Magnusson et al., 2005), it is also likely that the mechanism employed by Fzd9 to inhibit AChR clustering may somehow require the neuronal counterpart.

Wnt3a disassembles AChR clusters possibly through a β-catenin-dependent, but TCF-independent, signaling that results in the down-regulation of rapsyn (Wang et al., 2008). In addition, treatment of myotubes with lithium (Sharma and Wallace, 2003) or BIO (Henriquez et al., 2008), two GSK3β inhibitors that activate the Wnt/β-catenin pathway, inhibits agrin-induced AChR clustering. Consistent with these findings, our *in vitro* data show that Fzd9 accumulates β-catenin in myotubes and confirm previous results showing that Fzd9 activates a Wnt/β-catenin-dependent pathway (Karasawa et al., 2002). Remarkably, we found that β-catenin is present in the synaptic domain of embryonic NMJs. Together, our present findings support the view that Fzd9 inhibits AChR clustering by activating a β-catenin dependent Wnt pathway. In this regard, the role of β-catenin in NMJ formation has remained rather controversial. Genetic ablation of β-catenin in mouse skeletal muscles, but not in motor neurons, gives rise to abnormal NMJs that are distributed in wider end-plate bands than controls, an effect that was primarily related to pre-synaptic defects in axonal branching and neurotransmission (Li et al., 2008; Wang and Luo, 2008). In turn, specific stabilization of β-catenin in muscle, but not in neuronal cells, resulted in excessive nerve branching and defasciculation, while NMJ formation and function were unaffected (Liu et al., 2012). Interestingly, β-catenin deficient mice contain bigger AChR clusters than control animals (Li et al., 2008; Wang et al., 2008), further supporting the view that β-catenin signaling in muscle cells plays a negative role on NMJ formation and growth. However, in cultured muscle cells, the blockade of β-catenin interaction with α-catenin or the silencing of β-catenin expression results in impaired agrin-dependent AChR clustering (Zhang et al., 2007). These apparent discrepancies on the role of muscle β-catenin on AChR clustering could be explained by the ability of β-catenin to activate the canonical Wnt signaling cascade but also to play a key role in cell adhesion by forming a link between cadherins and the actin cytoskeleton (Brembeck et al., 2006). Whether Fzd9 accumulation of β-catenin exerts its inhibitory effects on AChR aggregation through any of these signaling pathways is still to be determined.

Complementing previous results showing that muscle Fzd9 expression relies on innervation (Magnusson et al., 2005), our present findings suggest that the specific localization of Fzd9 in developing NMJs, and the possible subsequent activation of a β-catenin dependent signaling that disassembles AChR clusters, could depend on the establishment of specifically located postsynaptic densities *in vivo*. As a general conclusion, our present findings support the notion that a fine balance between different Wnt pathways could contribute to shape the complex postsynaptic apparatus at the vertebrate NMJ.

### Conflict of interest statement

The authors declare that the research was conducted in the absence of any commercial or financial relationships that could be construed as a potential conflict of interest.
